# Mr. Chamberlain on the London School of Tropical Medicine

**Published:** 1905-05-20

**Authors:** 


### MR. CHAMBERLAIN ON THE LONDON SCHOOL OF TROPICAL MEDICINE.

"It is my duty and my pleasure to appeal to you with confidence, in the first place as men of business to contribute towards the greater safety and comfort of those workers to whom you owe your security in the pursuit of peaceable commerce. I appeal also to your national pride and Imperial patriotism. I call upon you to justify your claim to be the members of a great governing race and ask you to sympathise with those makers of your Empire who have already done so much to secure its renown and who have contributed and are contributing to the happiness and the welfare of the vast populations that you have gathered together under the protecting influence of a single flag."

In these words the Right Hon. Joseph Chamberlain concluded an eloquent appeal to the brilliant assembly of statesmen, scientists, and business men interested in the Tropics, who, to the number of about 500, had gathered together at the festival dinner in aid of the London School of Tropical Medicine, which took place at the Hôtel Cecil on Wednesday, the 10th inst.

After a brief introduction of the loyal toasts, Mr. Chamberlain proposed the toast of the evening, "The London School of Tropical Medicine." In doing so he said that he thought that those present would agree with him that attendance at a great public banquet of this kind or any inordinate prolongation of such festivities was inconsistent with the position of an advocate and supporter of preventive medicine. (Laughter.) Although on that account he had felt bound to decline numerous invitations from too hospitable friends, he had not been able to resist the temptation to repeat his experience of six years ago when he had presided at a similar banquet, the one at which that school was established. Now he came back to them, rejoiced at their past success, to assist them to provide for their future. It was a privilege, as well as a duty, to be in any way associated with that movement. They owed this duty to the vast populations for which they had gradually made themselves responsible, and they owed it still more to those of their own race who were daily risking health and life in order to maintain the honour and the interests of this country abroad. In the progress of the great work of discovery, which had disclosed secrets of geography hidden for centuries, discoveries which had linked up all parts of the world, those who had carried out their work with such skill and resolution, had suffered from our ignorance of the new conditions they would have to face and from our inability to deal with them. Indeed, the result had been that many of them had lost their health and their lives from this cause. It would be shameful if they allowed these sacrifices to continue for want of any preventive precautions on their side. He need not dilate on the advantages which must necessarily follow the establishment of such an institution as the London School of Tropical Medicine. They were still at the very beginning of its inquiries and of its operations, but what had been done showed how much it was probable they would have to be able to do in the future. The results they had achieved, for instance in regard to malaria, had been striking. In Cuba, too, they had succeeded in mitigating the dangers of yellow fever, and throughout West Africa the average of fatal cases from this scourge had been very much reduced. Expeditions of inquiry, too, had been sent out to make special inquiries, in some cases by the Liverpool School, in others by the London School, in co-operation with or under the direction of the Royal Society. Most of the tropical diseases which had hitherto played unrestricted havoc with the native populations, had been studied and their evils reduced. For most of what had been done they had to thank private enterprise. They had to thank Sir John Craggs for establishing travelling scholarships, and they also had to thank a Parsee gentleman, Mr. Bomanji Petit, for his gift of £7,000. (Applause.) But if they were grateful to these, they ought still more to be grateful to those who had taken part in the expeditions, not concealing from themselves what was involved. These expeditions were in many cases more dangerous than war, surely they were not less honourable. In carrying them out they had their casualties. First Mr. Myers, now more recently Dr. Dutton, both distinguished scientists in their way, both given up to the pursuit of this research, had fallen victims to their unselfishness. They were true martyrs to science. How were they to honour their memories? What statues or monuments should they erect to them? He thought no better expression could be given to our wish to commemorate their work than to support the thing for which they gave their lives. The London Committee asked for £100,000 to repay the debt, to make a proper provision for the teaching staff, and above all to subsidise research into those diseases indigenous to the Tropics. It seemed to him that there could not be found any more desirable application of superfluous wealth than to enable this institution to unite the work of the investigator and of the physician, which was after all the ideal union between scientific inquiry and practical application. (Applause.)

Sir Patrick Manson, in reply, expressed thanks on behalf of his colleagues and himself to Mr. Chamberlain for his presence that evening.

The Right Hon. Alfred Lyttelton proposed and Lord Strathcona responded to the toast of "The Empire."

Subscriptions amounting to over £10,000 were announced, and the proceedings terminated with the health of "The Chairman," proposed by the Duke of Marlborough.

